# Health Statistics in Australia: What We Know and Do Not Know

**DOI:** 10.3390/ijerph19094959

**Published:** 2022-04-19

**Authors:** Richard Madden, Nicola Fortune, Julie Gordon

**Affiliations:** 1 Faculty of Medicine and Health, University of Sydney, Sydney, NSW 2006, Australia; 2 Centre for Disability Research and Policy, University of Sydney, Sydney, NSW 2006, Australia; nicola.fortune@sydney.edu.au; 3 Centre of Research Excellence in Disability and Health, University of Melbourne, Carlton, VIC 3053, Australia; 4 WHO Collaborating Centre for Strengthening Rehabilitation Capacity in Health Systems, University of Sydney, Sydney, NSW 2006, Australia; julie.gordon@sydney.edu.au

**Keywords:** health statistics, Australian health system, health surveys, Indigenous, data linkage

## Abstract

Australia is a federation of six states and two territories (the States). These eight governmental entities share responsibility for health and health services with the Australian Government. Mortality statistics, including causes of death, have been collected since the late 19th century, with national data produced by the (now) Australian Bureau of Statistics (ABS) from 1907. Each State introduced hospital in-patient statistics, assisted by State offices of the ABS. Beginning in the 1970s, the ABS conducts regular health surveys, including specific collections on Aboriginal and Torres Strait Islander peoples. Overall, Australia now has a comprehensive array of health statistics, published regularly without political or commercial interference. Privacy and confidentiality are guaranteed by legislation. Data linkage has grown and become widespread. However, there are gaps, as papers in this issue demonstrate. Most notably, data on primary care patients and encounters reveal stark gaps. This paper accompanies a range of papers from expert authors across the health statistics spectrum in Australia. It is hoped that the collection of papers will inform interested readers and stand as a comprehensive review of the strengths and weaknesses of Australian health statistics in the early 2020s.

## 1. Introduction

Australia is a federation of six states and two self-governing territories (the Australian Capital Territory and the Northern Territory), referred to in this paper for simplicity as ‘the States’. These eight governmental entities share responsibility for health and health services with the Australian (Commonwealth) Government. Many health services are provided by governments, with the private sector also delivering services, notably in primary care, dentistry, private hospitals, and pharmacy. Health constitutes some 10% of the Australian economy [1] (p. 173).

Australia introduced a system of universal health insurance (now known as Medicare) in the 1970s and 1980s. This covered privately provided medical services and shared the funding of public hospitals between the Australian and State governments [2]. As a result, there was a need to know much more about the services that the Australian health system delivered across its many arms. The decades that followed have seen great progress in building a national health information infrastructure to inform health policy, resource allocation, and delivery of health care across the nation. That work continues.

This paper is a commentary that aims to briefly describe the main components of Australia’s health statistics system, highlight its breadth, successes, and novel features, point out some limitations, and indicate directions for future development. It provides a succinct overview of the current state of health statistics in Australia, set within a historical context, to inform future work to improve and build on Australia’s health information infrastructure, and to demonstrate the crucial role of health statistics in running an effective and responsive health system.

In preparing this commentary, we have drawn upon a broad range of reports, technical documents, and other resources available on the websites of Australia’s two main national statistics agencies (the Australian Bureau of Statistics and the Australian Institute of Health and Welfare) and the Australian Department of Health. The paper accompanies, and is informed by, a range of papers from expert authors across the health statistics spectrum in Australia. It is hoped that the collection of papers will inform interested readers and stand as a comprehensive review of the strengths and weaknesses of Australian health statistics in the early 2020s.

## 2. Australia’s Statistical Agencies

Health statistics in Australia come, in large part, from two official statistics agencies—the Australian Bureau of Statistics and the Australian Institute of Health and Welfare. Both agencies are apolitical and explicitly serve all sectors of the community. Their values accord with the UN Fundamental Principles of Official Statistics [3], which state, as Principle 1, ‘Official statistics provide an indispensable element in the information system of a democratic society, serving the Government, the economy and the public with data about the economic, demographic, social and environmental situation’. Principle 1 goes on to emphasise the need for impartiality and that statistics should be of ‘practical utility’.

The (now) Australian Bureau of Statistics (ABS) dates from 1905, as the national statistics agency. It conducts a population census every five years, processes and publishes vital statistics, and conducts a range of social surveys, including in health. In 1987, the (now) Australian Institute of Health and Welfare (AIHW) was established to focus on health and community services statistics, especially using administrative data provided by the States. The AIHW works in conjunction with the ABS. Both agencies operate under national legislation which strictly protects the confidentiality and privacy of individual data [4,5]. The AIHW is required to report to Parliament on the state of Australia’s health and health services every two years, in a publication called *Australia’s Health*, beginning in 1988, with the most recent in 2020 [1].

## 3. Development of Health Statistics

This paper gives a brief description of various categories of health statistics in Australia, beginning with an outline of developments from the beginning of the 20th century. Sources of health statistics include patient and administrative data, surveys, and clinical registries.

Mortality statistics, including causes of death, have been collected since the late 19th century, with national data produced by the ABS from 1907.

Each State established a hospital in-patient data collection by the 1970s. Data on each patient episode was provided by the hospital to the State central collection. On its establishment, the first task for the AIHW was to produce national hospital in-patient statistics. This was a vital need as, under Medicare, funding of hospitals was now shared by the Commonwealth and States.

To pursue its charter, to bring together State health data into national collections, the AIHW led the development of the National Health Information Agreement in 1992. Under this agreement, all States agreed to establish national minimum datasets for key services, including hospital in-patients, and provide annual data to the AIHW for collation and publication. National minimum dataset specifications were developed, data standards were published in the National Health Data Dictionary (available in electronic form from July 1997) and, in the early 2000s, AIHW established a national online metadata registry for health, housing, and community services statistics and information (METeOR) [6].

National Health Information Plans were developed in 1995 and 2002 [7,8], to identify agreed priorities for national developments in health statistics. Development of a new National Health Information Strategy began in 2019 [9] but was not completed before national health governance arrangements changed in the light of the COVID-19 pandemic. This paper refers to the priorities of the 2002 plan, many of which remain just as relevant today.

Australia follows international standards for data collection and analysis where these exist, notably for causes of death (World Health Organization (WHO)) [10] and health expenditure (Organisation for Economic Co-operation and Development (OECD)) [11]. The AIHW is the Australian Collaborating Centre for the WHO’s Family of International Classifications, the focus for Australian work on the development and maintenance of health classifications.

Australia’s health statistics are financed through a variety of arrangements, including national direct funding of AIHW and ABS, contract funding by Australian Government departments, and State health department funding for administrative data and some special-purpose collections.

## 4. Health Surveys

The ABS conducted its first National Health Survey in 1977–1978, and these surveys have been repeated at regular intervals. In 2011, a National Nutrition and Physical Activity Survey and a National Health Measures Survey were added, providing biomedical information, in addition to self-reported information on health conditions such as cardiovascular disease, diabetes, kidney function, and risk factors.

The first of now regular National Aboriginal and Torres Strait Islander Health Surveys was conducted in 2004 [12]. Some health data are also collected in National Aboriginal and Torres Strait Islander Social Surveys [13].

In addition to these national health surveys, the AIHW has conducted a regular National Drug Strategy Household Survey, beginning in 1985, gathering information on the use of alcohol, tobacco, and illicit drugs [14].

The ABS conducted a National Survey of Mental Health and Wellbeing in 2007, giving a one-off view of the characteristics of people with mental health conditions (employment, housing, etc.) [15].

In 2019–2020, the ABS conducted a Patient Experiences Survey, covering health service use and experiences with health providers, as part of its annual Multipurpose Household Survey [16].

## 5. Health Statistics for Aboriginal and Torres Strait Islander Peopless

Australia has generally high health status, but notably, Aboriginal and Torres Strait Islander (Indigenous) people experience disadvantages, compared with other Australians, across a range of health outcomes [17].

Up to 1988, statistics on the health of Australia’s Aboriginal and Torres Strait Islander people were almost non-existent. Australia became a nation in 1901. Its Constitution specified that ‘Aboriginal natives’ were not to be included in population estimates. This now-shocking provision ensured that there was little effort on statistics for the Indigenous population. Population estimates were conducted administratively, almost certainly underestimating the actual Indigenous population [18]. The exclusion provision was removed from the Constitution in 1967, and data about Indigenous status have been available since the 1971 Census, with Indigenous identification steadily improving over time [18].

In 1988, the first edition of AIHW’s biennial report, *Australia’s Health,* brought together an array of data to demonstrate the poor health status of Indigenous Australians.

The release of the first National Aboriginal and Torres Strait Islander Survey by the ABS in 1994 [19] marked a considerable step forward by producing a wide range of information on Indigenous people. The survey captured data on positive aspects of Indigenous life and culture, such as connection to land, as well as highlighting the systemic and inter-generational problems Indigenous people live with, including historical separation of children from families.

From 1996 on, the AIHW and ABS have worked in collaboration with Indigenous people to improve information on Indigenous health. A highlight was the release in 1997 of the first edition of *The Health and Welfare of Australia’s Aboriginal and Torres Strait Islander Peoples*, published jointly by the two agencies and launched by the Australian Governor-General [20].

The development of Indigenous health statistics was the number one priority of the 2002 National Health Information Plan [8].

The development of Indigenous health statistics in Australia and many challenging issues are described by Ring and Griffiths [21] in this issue. The continuing, nationally acknowledged but persistent ‘gap’ between the health of Indigenous people and that of other Australians is highlighted each year in national ‘Closing the Gap’ reports [22]. Indigenous health statistics will remain a priority for development and a focus for lively debate into the future. Indigenous people are increasingly leading the development of new Indigenous controlled data and pushing the national statistical agencies to redouble their efforts.

## 6. Mortality

Each Australian state requires registration of deaths that occur in that state, using a standard death certificate that is aligned with international requirements set by the World Health Organization. Data on causes of death have been recorded in line with the International Classification of Diseases (ICD) since 1907, with over 100 years of causes of death available for analysis.

In 2006, the AIHW published *Mortality over the twentieth century in Australia* [23]. This publication showed the path of key diseases over the 20th century. For example, the female death rate for cancer did not vary much over the century, at 150 deaths per 100,000 population, although the composition changed, with lung cancer rising sharply, while cancers of the stomach, cervix, and uterus fell. The male cancer death rate increased from 166 deaths per 100,000 population in 1907 to 287 in 1985 and then fell to 247 by the year 2000, with lung cancer being the major varying cause of death. The male death rate for circulatory diseases increased from 437 deaths per 100,000 population in 1907 to 1020 in 1968, before falling to 319 in 2000.

Cause-of-death coding for Australia is centralised at the ABS in Brisbane (using internationally developed automated software), facilitating the development of specialist skills. For the past 15 years, the ABS has worked closely with State registrars and the National Coronial Information System (NCIS), which collates causes of death for all deaths referred to coroners across Australia and New Zealand. One particular result of this collaboration is much more complete data on deaths by suicide. The ABS now revises causes of death where updated information becomes available from coronial investigations which can take several years, and deaths can be coded as suicides based on the information in the NCIS.

The paper by Eynstone-Hinkins and Moran [24] in this issue provides up-to-date information on Australian mortality statistics, including COVID-19 deaths.

## 7. Hospital Treatment

As already described, a national collection of hospital in-patient data was the first task of the AIHW on its establishment in 1987. A national minimum dataset was introduced in 1990. Annual data have been published since 1993–1994, and the reporting has become progressively more timely. The collection covers patients in public and private hospitals [25].

Statistics on emergency department presentations are also produced, including principal diagnosis and triage category, as well as demographic characteristics of patients.

Non-inpatient data remain limited to administrative characteristics, with no information yet available on reasons for encounter, diagnoses, or interventions.

Hospital in-patient data have formed the information base for many important health policy developments at national and state levels, including in relation to casemix funding for hospitals, potentially preventable hospitalisation, and quality and safety developments in hospitals. Additionally, equity issues around hospital access and variations in intervention patterns can be explored.

### 7.1. Casemix Funding

Casemix funding was developed at a national level from the 1980s and was first introduced in Victoria in 1993. Casemix is now referred to in Australia as activity-based funding. Australia adopted the casemix models originally developed in the United States [26]. Casemix is a measure of hospital output for each patient, based on their diagnoses and interventions provided.

The Australian casemix system for acute in-patients (Australian Refined Diagnosis Related Groups) is based on hospital in-patient statistics and a hospital costing survey. The classifications used are an Australian modification of ICD-10 for diagnoses (ICD-10-AM) and the Australian Classification of Health Interventions (ACHI). Supplementary systems exist for sub-acute patients such as rehabilitation and palliative care.

The Australian activity-based funding system, including its supporting classifications, is not in the public domain. Several countries have licences from the Australian Government to use the system in their countries.

### 7.2. Potentially Preventable Hospitalisation

Potentially preventable hospitalisation is an indicator of the effectiveness of primary care. There is a range of vaccine-preventable, acute, and chronic conditions for which policymakers believe hospitalisation could be prevented by earlier community-based care, particularly significant diagnoses of interest include complications of diabetes and chronic obstructive pulmonary syndrome (COPD) [1] (Section 5). This is an example where statistics from one sector of the health system can be used as a performance indicator for another sector.

### 7.3. Quality and Safety

Selected diagnoses relating to ‘hospital-acquired complications’ are used as indicators of quality and safety issues in Australian hospitals [27]. There are 16 complications, including pressure injuries, health-care-associated infections, and respiratory complications. Hospital funding arrangements now include an adjustment for hospital-acquired complications, taking into account the non-preventable occurrences of these conditions.

Regrettably, there is duplication of hospital in-patient data collections. The Independent Hospitals Pricing Authority collects data from the States for its activity-based funding (casemix) functions. The Australian Department of Health collects clinical, demographic, and financial information for privately insured in-patients from private health insurers, and, through the Australian Private Hospital Data Bureau, also collects data from private hospitals covering patient demographics, clinical information, and hospital charges. The AIHW’s collection covers most of the data items collected in these collections, apart from information on charges to private patients.

## 8. Primary Health Care

In contrast to information on hospital in-patients, primary health care statistics have had a chequered history and are a significant weak point in Australian health statistics. Primary health care practitioners, including general practitioners (GPs), nurses, allied health professionals, pharmacists, dentists, and Aboriginal and Torres Strait Islander health workers, provide services in a range of community settings and are critical first points of contact with the health system.

In 1998, a national sample-based data collection of general practice encounters (BEACH) was put in place by the University of Sydney and the AIHW, with wide-ranging support from general practitioners’ professional representative groups and in partnership with a number of pharmaceutical companies. This unique partnership offered a publicly accessible dataset and provided information to participating pharmaceutical companies on their products’ uses. The paper by Gordon et al. [28] in this issue outlines the development and uses of this data collection.

The Australian Government terminated funding for the BEACH collection in 2016 at short notice and without an alternative data collection in place. Currently, general practice statistical data are limited to extracts from GPs’ electronic records, which come from multiple systems without any common data architecture or standards [29]. The samples drawn are not always structured to enable the production of statistics about all elements of the population, especially those with significant health disadvantages, such as Indigenous Australians. The AIHW is leading the development of a National Primary Health Care Data Asset [30] of which statistics about general practice are one component.

## 9. Public Health

In 1999, a National Public Health Information Plan was published, which focused on the need for improving national health surveys. As a result, information on a range of risk factors has been expanded and systematised over the past 20 years—smoking, alcohol use, exercise, and diet are examples.

The *Australia’s Health* series reports on a range of population health indicators, as well as information on national screening programs. Between 2010 and 2013, Australia had a specialist agency focusing on preventive health. It produced a national report, *State of Preventive Health 2013* [31], which has not been repeated. This report brought together the national data on major risk factors, as well as international comparisons.

The National Notifiable Diseases Surveillance System brings together reports on notifiable diseases across Australia. Fortnightly reports are produced. Notifications in respect of Indigenous people made up more than half of the 330,000 notifications in 2016, the latest report available [32].

COVID-19 has seen separate and timely reporting of data on infection cases, hospitalisations, and deaths. It remains to be seen whether this results in the quality and timeliness of notifiable diseases information being improved and strengthened.

## 10. Mental Health

The paper in this issue by Rosenberg et al. [33] describes the development of mental health statistics since the 1990s. This development occurred outside the processes established under the National Health Information Agreement, even though the Australian Government and State health agencies were cooperating through their mental health experts. A separate governance arrangement was established through the Mental Health Information Strategy Sub-Committee.

The AIHW publishes an annual review of mental health services and associated resources in Australia [34,35]. Data are drawn from across AIHW data collections and other sources, including the 2007 ABS National Survey of Mental Health and Wellbeing.

## 11. Medicines: Use and Outcomes

Comprehensive data on medicines provided to Australians in the community are available from the national Pharmaceutical Benefits Scheme (PBS), established in 1948. Medicines below the cost threshold for the PBS and those provided to public hospital in-patients are not included in the PBS.

The use of these data for pharmaco-epidemiological purposes is described by Pearson et al. [36] in this issue. Increasingly, medicines data are linked to other national datasets (see Section 15, Data Linkage, below). The authors note that studies in Australia are relatively few and do not utilise all of the datasets available; they explore possible paths to facilitate a leap forward in medicine outcome studies.

## 12. Data on Health and Health Disadvantage for Particular Population Groups

There have been several references already in this paper to health statistics concerning Indigenous Australians. There are other population groups whose health status and access to health services also need to be monitored, as they experience significant disadvantages in relation to health.

People with disability are one such group. Statistics on people with disability and disability support services have been greatly improved over the past 20 years, although the introduction of the National Disability Insurance Scheme, itself a major social reform, has led to a break in the series of nationally consistent data on disability services, which was collected through the Disability Services National Minimum Dataset from 1991 to 2019 [37]. However, information on the health of people with disability and their access to health services has generally come from health and disability surveys, rather than from health services statistics. The paper by Fortune et al. [38] in this issue discusses this in more detail.

Medicare, Australia’s universal health insurance system, gives all Australians the capacity to access high-quality medical and hospital services. The reality is that there is a clear excess burden of disease for lower socio-economic groups, notably in coronary heart disease, lung cancer, chronic kidney disease, and COPD [39]. In addition to survey evidence, data linkage is facilitating the examination of socio-economic variables in relation to health. For example, the ABS now links mortality data and census records, which has allowed examination of mortality due to various health conditions according to household equivalised income, highest educational attainment, and housing tenure [40].

Australia has about a quarter of its population born overseas, and almost half have at least one parent born overseas [41] (p. 271). Many health data collections include country of birth and language spoken at home. However, the AIHW has acknowledged that information on culturally and linguistically diverse (CALD) populations is among Australia’s data gaps [1] (p. 6). *Australia’s Health 2020* omitted data on CALD populations altogether. The 2018 edition briefly discussed the generally lower age-standardised mortality rates and rates of potentially preventable hospitalisations for people born outside Australia, compared with the Australian-born population. These data gaps have been thoroughly addressed in a recent report by the Federation of Ethnic Communities Councils of Australia (FECCA) [42].

In Australia, prisons and corrective services are the responsibility of the States. Without Australian Government involvement, it took many years for a national effort to report on the health of prisoners, which is the responsibility of State health departments or State correctional services agencies. Since 2009, the AIHW has conducted the National Prisoner Health Data Collection every 3 years. Data reported highlight significant mental health problems, high rates of smoking and drug use, and a high prevalence of disability among prisoners [43].

## 13. Health Registries

Each Australian State has operated a cancer registry for many years. The AIHW maintains the National Cancer Statistics Clearing House (NCSCH), which was established in 1986 as the national repository of cancer incidence and mortality statistics. The repository is used to produce national cancer statistics. Each jurisdiction uses the national minimum dataset for its reporting. In addition, the jurisdictions collaborate with the AIHW to produce registries for breast, cervical, and bowel cancer screening.

There is now a wide range of clinical registries in Australia. These include clinical quality, disease, immunisation, and product registries. A Framework for clinical quality registries has been developed by the Australian Commission on Safety and Quality in Health Care [44].

## 14. Oral Health

Dental statistics have been well developed in Australia through a specialist centre at the University of Adelaide, which has worked in collaboration with AIHW. Foundation work in South Australia was built to give a rich picture of child and adult dental health, as well as the dental health of Aboriginal and Torres Strait Islander peoples. The paper in this issue by Amarasena et al. [45] describes a recent national oral health survey and the changes in dental health over the past 30 years.

## 15. Data Linkage

Data linkage has been mentioned earlier in this paper. Data linkage involves the development of enriched datasets by linking two or more datasets. Data linkage in Australia commenced in Western Australia in the 1990s and now occurs in all State jurisdictions, and is supported by the Public Health Research Network (see Smith et al. in this issue) [46]. Ethical approval is essential for data linkage because linked data can readily produce identifiable data even if the original datasets are de-identified.

The AIHW, as described by Jensen [47] has developed the National Integrated Health Services Information Analysis Asset (NIHSIAA) linking a range of its datasets and other Australian Government datasets, thus bringing together data covering hospitals, Medicare, Pharmaceutical Benefits Scheme, Repatriation Pharmaceutical Benefits Scheme, residential aged care, and the National Death Index.

## 16. Financing

Australia has produced estimates of national health expenditure since 1980, following the OECD’s guidelines. The paper by Goss [48] in this issue discusses this work and presents a fascinating dissection of the growth in Australia’s health expenditure this century.

## 17. Workforce

From its commencement in 1987, the AIHW collated health workforce data from state registration authorities, with a focus on the medical and nursing workforce. At registration (then administered at the state level), individual practitioners were asked to provide demographic and employment information about themselves. The resulting statistics formed a valuable basis for workforce planning, highlighting urban/rural disparities in the medical workforce and the ageing of the nursing workforce.

In 2010, national health workforce registration was introduced through the National Registration and Accreditation Scheme, and responsibility for statistical reports remained with AIHW. In 2016, responsibility for workforce statistics was passed to the Australian Department of Health [44].

The AIHW produced comprehensive reports on the health and community services workforces after the 1996, 2001, and 2006 population censuses [49].

## 18. Discussion

The Australian health system encompasses a mix of Australian Government and State Government responsibilities and is a combination of public and private services. This complexity makes a national health statistics system essential if the Australian health sector is to be understood, accountable, responsive, and improved. Since the 1980s, this system has been established, developed, and maintained. All jurisdictions and sectors have contributed to this effort. The 1992 National Health Information Agreement (NHIA) provided a critical framework for the development of national datasets, ensuring common data standards have been adopted in these datasets. The contrast with health sectors that have stayed outside the NHIA arrangements is stark.

Australia now has a comprehensive array of health statistics, published regularly without political or commercial interference. Privacy and confidentiality are guaranteed by legislation.

However, there are gaps, as some papers in this issue illustrate; most notable are data on primary care patients and encounters, with no current reliable information on the reason for encounter, consultation outcome, and other aspects of primary health care. Similar gaps exist for patients treated by medical specialists outside hospitals, and for patients of allied health practitioners. Additionally, some datasets (such as health workforce) exist in silos, separate from the national statistical agencies where users would expect to find information readily accessible.

The utility of national health statistical collections is dependent on the development and widespread use of national minimum datasets, which ensure the supply of comparable data from different sectors (such as public and private hospitals) and jurisdictions. The more recent emergence of ‘big data’ sets and analysis provides new opportunities, as long as good statistical practices are followed [50] and high ethical and privacy standards are adhered to.

The papers in this issue highlight that health statistics must respond to health policy needs and developments, and to emerging health issues. Casemix funding for hospitals energised the development and supply of hospital statistics and now relies on them. COVID-19 has led to more timely incidence and mortality statistics and focused attention on the calculation of excess mortality in a pandemic [35].

Work on the postponed National Health Information Strategy should be resumed so that clear priorities for health statistics developments are identified and committed to by all stakeholders. National consultation had occurred prior to the deferral of the development in 2020, which naturally gave rise to wide-ranging demands for improved data and analysis. It is important that the strategy focuses on a few key areas with clear short- and medium-term priorities. These include the following aspects:

- Primary health care: Broad-ranging work on a National Primary Health Care Data Asset has been underway for some years by the AIHW. This appears to have an ambitious scope and needs to be seen as a project of long-term development. Immediate steps are needed to fill the gap left by the termination of the BEACH collection, with a robust, statistically reliable, and nationally representative collection. The limitations of generating statistics based on data extraction from GP electronic records must be acknowledged and addressed, as well as methods developed to overcome these.
- Disability: The development of a National Disability Data Asset is advancing, and significant funding was allocated by the Australian Government in late 2021. This development is broadly focused and relies mainly on the identification of people with disability through disability-specific services and payments. The ability to identify people with disability consistently within health service data systems is necessary for monitoring equity of access and outcomes. Creating a succinct question or short set of questions that can function as a disability ‘identifier’ for use in administrative data collections is a key priority.
- Mental health: Developments in this sector have occurred outside the mainstream structures under the National Health Information Agreement, so statistics in this crucial health sector remain separate from other health statistics. Data linkage provides a strong platform today to bring together data on services provided in the various sectors: primary health care, community care, and hospitals. The National Health Information Strategy development needs to prioritise mental health statistics and integrate them with other health statistics streams.
- Hospital in-patient statistics: The duplication of collections described above should be removed, with the national collection for public and private hospitals managed by the AIHW, which should prepare a common dataset for all other national agencies. While some additional data items would need to be added to the AIHW collection, one collection would replace four.

## 19. Conclusions

Australia has a robust and reliable set of health statistics. This is the result of many decades of national governance cooperation, resourcing, and effort. The ongoing commitment of resources and collaboration among stakeholders continues to be essential to ensure a robust evidence base to inform policy and practice, based on nationally consistent data standards, and to underpin research efforts, into the future. Some specific potential improvements have been highlighted, and the full potential of data linkage has yet to be achieved.

People provide information about themselves and their health in many settings and are often unaware that this information provides input to health statistics which, in turn, improves health and health services [47]. Respecting individuals’ data remains at the heart of the health statistics effort, and using it as well as possible is a key responsibility for the statistical community.

## Data Availability

Not applicable.
